# On chaos control of nonlinear fractional Newton-Leipnik system via fractional Caputo-Fabrizio derivatives

**DOI:** 10.1038/s41598-023-49541-z

**Published:** 2023-12-20

**Authors:** Najat Almutairi, Sayed Saber

**Affiliations:** 1 Department of Mathematics, College of Science, Qassim University, Buraidah, Saudi Arabia; 2 Department of Mathematics and Statistics, Faculty of Science, Beni-Suef University, Beni Suef, Egypt; 3 Department of Mathematics, Al-Baha University, Baljurashi, Saudi Arabia

**Keywords:** Fractional derivatives, Nonlinear equations, Simulation, Numerical results, Iterative method, Time varying control system, Lyapunov functions, Engineering, Mathematics and computing

## Abstract

In this work, we present a design for a Newton-Leipnik system with a fractional Caputo-Fabrizio derivative to explain its chaotic characteristics. This time-varying fractional Caputo-Fabrizio derivative approach is applied to solve the model numerically, and to check the solution’s existence and uniqueness. The existence and uniqueness of results of a fractional-order model under the Caputo-Fabrizio fractional operator have been proved by fixed point theory. As well, we achieved a stable result by applying the Ulam-Hyers concept. Chaos is controlled by linear controllers. Furthermore, the Lyapunov exponent of the system indicates that the chaos control findings are accurate. Based on weighted covariant Lyapunov vectors we construct a background covariance matrix using the Kaplan-Yorke dimension. Using a numerical example, this suggested method is illustrated for its applicability and efficiency.

## Introduction

Chaos control research has significant application potential in many fields, especially communication, electric power, computing, and medicine. Due to their numerous physical and circuit modeling applications to chaotic systems, they have attracted numerous researchers. In 1981, Leipnik and Newton studied their concept of the gyroscope for chaotic motion, discovering two strange attractors for rigid bodies’ motion. Wang and Tian named this system the Newton-Leipnik system^1^. Since Leipnik and Newton’s work, many scientists have intensively examined the chaotic dynamics of rigid body motion^2^-^11^. In addition, Chen and Lee^3^ introduced a novel chaotic system capable of generating dual-role chaotic attractors when investigating rigid body motion anti-chaos control. Richter^12^ studied the stability and chaos control of Newton-Leipnik systems using static nonlinear feedback laws based on Lyapunov functions. The Newton-Leipnik system^13^ with two strange attractors is described by the ordinary differential equation:

1 $$\begin{aligned} \begin{aligned} \dot{{\text {x}}}({\text {t}})&=-a{\text {x}}({\text {t}})+{\text {y}}({\text {t}})+10{\text {y}}({\text {t}}){\text {z}}({\text {t}}), \\ \dot{{\text {y}}}({\text {t}})&=-{\text {x}}({\text {t}})-0.4{\text {y}}({\text {t}})+5{\text {x}}({\text {t}}){\text {z}}({\text {t}}),\\ \dot{{\text {z}}}({\text {t}})&= b{\text {z}}({\text {t}})-5{\text {x}}({\text {t}}){\text {y}}({\text {t}}). \end{aligned} \end{aligned}$$

Recent years have seen an increase in research on the chaotic dynamics of Caputo-Fabrizio, fractional operators, etc., see for example^14–49^. The Caputo-Fabrizio fractional derivatives have a non-singular kernel which describes various processes accurately^50,51^. In this paper, we present a generalized numerical scheme for fractional Caputo-Fabrizio kernels for Newton-Leipnik systems and use a simple linear controller to control it. The implicit solutions to the problem are derived, and the solutions under different fractional orders are compared intuitively through images. Furthermore, variable-order Newton-Leipnik models can better explain the memory and genetic properties of equations (1). The Lyapunov exponent, bifurcation diagram, and other phase diagrams of the time-varying fractional Newton-Leipnik system to assess the impact of modifying the derivative order and parameter values are investigated. A straightforward linear controller regulates chaos, and a numerical simulation is produced. To demonstrate the applicability and efficacy of this novel strategy, we provide a numerical example.

The novelty of this paper lies in the following: Due to the high non-linearity of our problem, we used a suitable numerical scheme to solve this system of equations numerically. The Caputo-Fabrizio fractional derivatives have a non-singular kernel which describes various processes accurately. The implicit solutions to the problem are obtained, and the solutions under different fractional orders are compared intuitively through images. By comparing the results obtained in this paper with those achieved under Caputo fractional derivatives, it is found that the solutions change relatively gently under Caputo-Fabrizio fractional derivatives. Additionally, the differential equation calculation under the Caputo-Fabrizio derivative is relatively simple and convenient, which is not the case with other fractional derivatives. Using linear control, we have introduced the methodology for synchronizing Newton-Leipnik systems with fractional Caputo-Fabrizio derivatives. Very few or no researchers investigated this result. So, this is the novelty of this manuscript.

The remainder of the paper is organized as follows: section “Introduction”, following the introduction. A Newton-Leipnik system in the sense of Caputo-Fabrizio fractional derivatives is formulated in section “Model analysis” and its existence and uniqueness are established. Furthermore, a detailed analysis of the stability, Lyapunov exponent (LE) and Kaplan-Yorke dimension of this system has been obtained in the same section. The control of the proposed system (2) in the sense of Caputo-Fabrizio has been discussed in section “Kaplan-Yorke dimension”. In section “Control of Newton-Leipnik systems”, numerical schemes are applied to solve the developed model, and numerical simulations are conducted to verify the analytical findings. In addition, this section summarizes the major findings of the study and discusses their behavior.

## Model analysis

### Model formulation

#### Definition 1

Let $u: \mathbb {R}^{+} \rightarrow \mathbb {R}$. The left Caputo fractional derivative of fractional order $\alpha$ of the function *u*(*t*) is defined by;

$$\begin{aligned} {}^{{\text {C}}} \mathscr {D}_{{\text {t}}}^{\alpha } u(t)=\frac{1}{\Gamma (n-\alpha )} \int _{0}^{t}(t-s)^{n-\alpha -1} u^{(n)}(s) d s,\quad t>0, \end{aligned}$$

where $\alpha \in (n-1, n), n \in \mathbb {N}$ and $\Gamma (z)=\int _0^\infty e^{-t}t^{z-1}dt$ is the Euler gamma function.

#### Definition 2

^50,51^ Let *u*(*t*) be continuous and differentiable on $C^{1}[0,1]$, then the Caputo-Fabrizio derivative with fractional order $\alpha$ of the function *u*(*t*) is given as follows

$$\begin{aligned} {}^{{\text {CF}}} \mathscr {D}_{{\text {t}}}^{\alpha } u(t)=\frac{M(\alpha )}{1-\alpha } \int _{0}^{t} \frac{d u(s)}{d \tau } \exp \left( \frac{-\alpha (t-s)}{1-\alpha }\right) d s, \end{aligned}$$

where $M(\alpha )$ is a normalization function such that $M(0)=M(1)=1$ and $0<\alpha \le 1$.

#### Definition 3

The Caputo fractional integral of the function *u*(*t*) is given as follows;

$$\begin{aligned} {}^{{\text {C}}} I_{t}^{\alpha }[u(t)]=\frac{1}{\Gamma (\alpha )} \int _{0}^{t} u(s)(t-s)^{\alpha -1} d s. \end{aligned}$$

#### Definition 4

^50,51^ The Caputo-Fabrizio fractional integral of the function *u*(*t*) is in the form of

$$\begin{aligned} {}^{{\text {CF}}} I_{{\text {t}}}^{\alpha }[u(t)]=\frac{1-\alpha }{M(\alpha )} u(t)+\frac{\alpha }{M(\alpha )} \int _{0}^{t} u(s) d s. \end{aligned}$$

Thus, the time-varying fractional Newton-Leipnik system is introduced as follows.

2 $$\begin{aligned} \begin{aligned} {}^{{\text {CF}}} \mathscr {D}_{{\text {t}}}^{\alpha } {\text {x}}({\text {t}})&=-a{\text {x}}({\text {t}})+{\text {y}}({\text {t}})+10{\text {y}}({\text {t}}){\text {z}}({\text {t}}), \\ {}^{{\text {CF}}} \mathscr {D}_{{\text {t}}}^{\alpha } {\text {y}}({\text {t}})&=-{\text {x}}({\text {t}})-0.4{\text {y}}({\text {t}})+5{\text {x}}({\text {t}}){\text {z}}({\text {t}}),\\ {}^{{\text {CF}}} \mathscr {D}_{{\text {t}}}^{\alpha }{\text {z}}({\text {t}})&=b{\text {z}}({\text {t}})-5{\text {x}}({\text {t}}){\text {y}}({\text {t}}). \end{aligned} \end{aligned}$$

### Existence and uniqueness

This section is the same as^20,21^, using Picard-Lindelof method and Banach fixed point theorem to prove the uniqueness of the solution of (2). Consider the following function:

$$\begin{aligned} \begin{aligned} \Psi _1({\text {x}}, {\text {y}},{\text {z}}, {\text {t}})&=-a{\text {x}}({\text {t}})+{\text {y}}({\text {t}})+10{\text {y}}({\text {t}}){\text {z}}({\text {t}}), \\ \Psi _2({\text {x}}, {\text {y}},{\text {z}}, {\text {t}})&=-{\text {x}}({\text {t}})-0.4{\text {y}}({\text {t}})+5{\text {x}}({\text {t}}){\text {z}}({\text {t}}), \\ \Psi _3({\text {x}}, {\text {y}},{\text {z}}, {\text {t}})&= b{\text {z}}({\text {t}})-5{\text {x}}({\text {t}}){\text {y}}({\text {t}}). \end{aligned} \end{aligned}$$

The Lipschitz continuity condition with Lipschitz constants arises from the following considerations. Assuming *u*, *v* and *w* are all bounded, we have

$$\begin{aligned} \begin{aligned} \bigl \Vert \Psi _1({\text {x}}_1, {\text {y}},{\text {z}}, {\text {t}})-\Psi _1({\text {x}}_2, {\text {y}},{\text {z}}, {\text {t}})\bigr \Vert&=\bigl \Vert -a{\text {x}}_1+{\text {y}}+10{\text {y}}{\text {z}} +a{\text {x}}_2-{\text {y}}-10{\text {y}}{\text {z}} \bigr \Vert \\&\le a \Vert {\text {x}}_{2}-{\text {x}}_{1}\Vert . \end{aligned} \end{aligned}$$

This proves that $\Psi _1({\text {x}}, {\text {y}},{\text {z}}, {\text {t}})$ is Lipschitz continuous, that is, in particular, the function $\Psi _1({\text {x}}, {\text {y}},{\text {z}}, {\text {t}})$ is bounded; that is, $\exists k_{1}$ such that $\Vert \Psi _1({\text {x}}, {\text {y}},{\text {z}}, {\text {t}}) \Vert \le k_{1}$. Use the $\Psi _1({\text {x}}, {\text {y}},{\text {z}}, {\text {t}})$ function to construct a Picard operator with fractional integration.

$$\begin{aligned} \begin{aligned} \mathscr {P}{\text {x}}({\text {t}})= {\text {x}}(0)+^{{\text {CF}}} I^{\alpha }_{{\text {t}}} \Psi _1({\text {x}}, {\text {y}},{\text {z}}, {\text {t}}). \end{aligned} \end{aligned}$$

Therefore, $$\exists \ell _1\in \mathbb {R}$$, $${\text {t}} \le \ell _1$$, satisfies

$$\begin{aligned} \begin{aligned} \bigl \Vert \mathscr {P}{\text {x}}({\text {t}}) - {\text {x}}(0) \bigr \Vert&= \bigl \Vert ^{{\text {CF}}} I^{\alpha }_{{\text {t}}} \Psi _1({\text {x}}, {\text {y}},{\text {z}}, {\text {t}}) \bigr \Vert \\&\le ^{{\text {CF}}} I^{\alpha }_{{\text {t}}} \bigl \Vert \Psi _1({\text {x}}, {\text {y}},{\text {z}}, {\text {t}}) \bigr \Vert \\&\le k_{1} ^{{\text {CF}}} I^{\alpha }_{{\text {t}}} (1)\\&\le \frac{2k_{1}}{\Lambda (\alpha )} \biggl ( \frac{1-\alpha +\ell _1\alpha }{2-\alpha }\biggr ). \end{aligned} \end{aligned}$$

This proves the boundedness of operator $\mathscr {P}$.

We have now given the condition for the operator $\Psi _1({\text {x}}, {\text {y}},{\text {z}}, {\text {t}})$ to be a contraction. To provide this condition, we use the following process:

$$\begin{aligned} \begin{aligned} \bigl \Vert \mathscr {P}{\text {x}}_{1}({\text {t}}) - \mathscr {P}{\text {x}}_{2}({\text {t}}) \bigr \Vert&= \bigl \Vert ^{{\text {CF}}} I^{\alpha }_{{\text {t}}} \Psi _1({\text {x}}_1, {\text {y}},{\text {z}}, {\text {t}})-^{{\text {CF}}} I^{\alpha }_{{\text {t}}} \Psi _1({\text {x}}_2, {\text {y}},{\text {z}}, {\text {t}}) \bigr \Vert \\&\le \bigl \Vert \Psi _1({\text {x}}_1, {\text {y}},{\text {z}}, {\text {t}}) - \Psi _1({\text {x}}_2, {\text {y}},{\text {z}}, {\text {t}}) \bigr \Vert ^{{\text {CF}}} I^{\alpha }_{{\text {t}}} (1)\\&\le a \bigl \Vert {\text {x}}_{1}({\text {t}}) - {\text {x}}_{2}({\text {t}}) \bigr \Vert ^{{\text {CF}}} I^{\alpha }_{{\text {t}}} (1)\\&\le \frac{2a}{\Lambda (\alpha )} \biggl ( \frac{1-\alpha +\ell _1\alpha }{2-\alpha }\biggr ) \bigl \Vert {\text {x}}_{1}({\text {t}}) - {\text {x}}_{2}({\text {t}}) \bigr \Vert . \end{aligned} \end{aligned}$$

Thus, $\mathscr {P}$ is a contraction map if:

$$\begin{aligned} \frac{2}{\Lambda (\alpha )} \biggl ( \frac{1-\alpha +\ell _1\alpha }{2-\alpha }\biggr ) \le \frac{1}{a}. \end{aligned}$$

So the solution of system (2) exists and is unique. According to the following proof, the solution is unique. Consider the two solutions ${\text {x}}_1({\text {t}})$ and ${\text {x}}_2({\text {t}})$ to the first equation of (2).

$$\begin{aligned} {\text {x}}_{1}({\text {t}})-{\text {x}}_{1}(0)= {^{{\text {CF}}}} I^{\alpha }_{{\text {t}}} \Psi _1({\text {x}}_1, {\text {y}},{\text {z}}, {\text {t}}),\\{\text {x}}_{2}({\text {t}})-{\text {x}}_{2}(0)= {^{{\text {CF}}}} I^{\alpha }_{{\text {t}}} \Psi _1({\text {x}}_2, {\text {y}},{\text {z}}, {\text {t}}). \end{aligned}$$

Taking the following difference and using the traingle inequality:

3 $$\begin{aligned} \begin{aligned} \bigl \Vert {\text {x}}_{1}({\text {t}}) - {\text {x}}_{2}({\text {t}}) \bigr \Vert&= \bigl \Vert ^{{\text {CF}}} I^{\alpha }_{{\text {t}}} \Psi _1({\text {x}}_1, {\text {y}},{\text {z}}, {\text {t}})- ^{{\text {CF}}} I^{\alpha }_{{\text {t}}}\Psi _1({\text {x}}_2, {\text {y}},{\text {z}}, {\text {t}}) \bigr \Vert \\&\le ^{{\text {CF}}} I^{\alpha }_{{\text {t}}} \bigl \Vert \Psi _1({\text {x}}_1, {\text {y}},{\text {z}}, {\text {t}})- \Psi _1({\text {x}}_2, {\text {y}},{\text {z}}, {\text {t}}) \bigr \Vert \\&\le \frac{2a}{\Lambda (\alpha )} \biggl ( \frac{1-\alpha +\ell _1\alpha }{2-\alpha }\biggr ) \bigl \Vert {\text {x}}_{1}({\text {t}}) - {\text {x}}_{2}({\text {t}}) \bigr \Vert . \end{aligned} \end{aligned}$$

So, from (3), one obtains the following relationship:

$$\begin{aligned} \begin{aligned} \bigl \Vert {\text {x}}_{1}({\text {t}}) - {\text {x}}_{2}({\text {t}}) \bigr \Vert \biggl [ 1 - \frac{2a}{\Lambda (\alpha )} \biggl ( \frac{1-\alpha +\ell _1\alpha }{2-\alpha }\biggr ) \biggr ] \le 0. \end{aligned} \end{aligned}$$

This leads to $\Vert {\text {x}}_{1}({\text {t}}) - {\text {x}}_{2}({\text {t}}) \Vert \le 0,$ so ${\text {x}}_{1}({\text {t}}) = {\text {x}}_{2}({\text {t}})$. We conclude that the first equation of (2) has a unique solution. Similarly, we can further prove the existence and uniqueness of the solution to the second and the third equation of (2). We conclude that the third equation of (2) has a unique solution.

## Stability analysis

### Equilibrium points

To compute the equilibrum points of the Newton-Leipnikk model (2), consider the following:

$$\begin{aligned} \begin{aligned} -au+v+10vw=0, \\ -u-0.4v+5uw=0,\\ bw-5uv=0. \end{aligned} \end{aligned}$$

Thus

$$\begin{aligned} \begin{aligned} J(E^{*})= \begin{pmatrix} -a &{} 1+10w^* &{} 10v^* \\ -1+5w^* &{} -0.4 &{} 5u^* \\ -5v^* &{} -5u^* &{}b \end{pmatrix}. \end{aligned} \end{aligned}$$

All equilibrium points are saddle points and satisfy the system chaos condition.

**Table 1:** Equilibrium points with related eigenvalues.

Equilibrium points	Eigenvalues	Nature	Index
$E_0=(0,0,0)$	0.175, $-\,0.4 \pm i$	Saddle-focus point	1
$E_1=(-\,0.2390,-\,0.0308,0.2103)$	$-\, 0.8, 0.0875 \pm 1.2113i$	Saddle-focus	2
$E_2=(-\,0.0315,0.1224,-\,0.1103)$	$- \,0.8, 0.0875 \pm 0.8752i$	Saddle-focus	2
$E_3=(0.0315,-\,0.1224,-\,0.1103)$	$-\, 0.8, 0.0875 \pm 0.8752i$	Saddle-focus	2
$E_4=(0.2390,0.0308,0.2103)$	$-\, 0.8, 0.0875 \pm 1.2113i$	Saddle-focus	2

### Hyers-Ulam stability

The Hyers-Ulam stability has been motivated by the work done in  [32, 33]^29,30^

#### Definition 5

The constants $\zeta _i>0$, for $i \in \mathbb {N}_{1}^{3}$ must meet the following conditions for model (2) to have Hyers-Ulam stability:

$$\begin{aligned}\Big |{\text {x}}(\texttt{t})- {\frac{1-\alpha }{M(\alpha )}}\Psi _1(\texttt{t},\Psi (\texttt{t})) -{\frac{\alpha }{M(\alpha )}} \int _{0}^\texttt{t}\Psi _1(\xi ,\Psi (\xi ))d\xi \Big | \le \zeta _{1},\\\Big |{\text {y}}(\texttt{t})- {\frac{1-\alpha }{M(\alpha )}}\Psi _2(\texttt{t},\Psi (\texttt{t})) -{\frac{\alpha }{M(\alpha )}} \int _{0}^\texttt{t}\Psi _2(\xi ,\Psi (\xi ))d\xi \Big | \le \zeta _{2},\\\Big |{\text {z}}(\texttt{t})- {\frac{1-\alpha }{M(\alpha )}}\Psi _3(\texttt{t},\Psi (\texttt{t})) -{\frac{\alpha }{M(\alpha )} }\int _{0}^\texttt{t}\Psi _3(\xi ,\Psi (\xi ))d\xi \Big | \le \zeta _{3}. \end{aligned}$$

In the model (2), an approximation is $\Big ({\text {x}}_1(\texttt{t}), {\text {y}}_1(\texttt{t}), {\text {z}}_1(\texttt{t})\Big )$ which satisfies the following:

$$\begin{aligned} \begin{aligned} {\text {x}}_1(\texttt{t})&=\frac{1-\alpha }{M(\alpha )}\Psi _1(\texttt{t},{\text {x}}_1(\texttt{t})) +\frac{\alpha }{M(\alpha )} \int _{0}^\texttt{t}\Psi _1(\xi ,{\text {x}}_1(\xi ))d\xi ,\\ {\text {y}}_1(\texttt{t})&=\frac{1-\alpha }{M(\alpha )}\Psi _2(\texttt{t},{\text {y}}_1(\texttt{t})) + \frac{\alpha }{M(\alpha )} \int _{0}^\texttt{t}\Psi _2(\xi ,{\text {y}}_1(\xi ))d\xi ,\\ {\text {z}}_1(\texttt{t})&=\frac{1-\alpha }{M(\alpha )}\Psi _3(\texttt{t},{\text {z}}_1(\texttt{t})) + \frac{\alpha }{M(\alpha )} \int _{0}^\texttt{t}\Psi _3(\xi ,{\text {z}}_1(\xi ))d\xi , \end{aligned} \end{aligned}$$

so that

4 $$\begin{aligned} \begin{aligned} \Big |{\text {x}}-{\text {x}}_1\Big |&\le {\text {v}}_{1} \omega _{1}, \\ \Big |{\text {y}}-{\text {y}}_1\Big |&\le {\text {v}}_{2} \omega _{2}, \\ \Big |{\text {z}}-{\text {z}}_1\Big |&\le {\text {v}}_{3} \omega _{3}. \end{aligned} \end{aligned}$$

#### Theorem 1

If (4) is true, then model (2) has Hyers-Ulam stability.

#### Proof

$$\begin{aligned} \begin{aligned} \Big |{\text {x}}-{\text {x}}_1\Big |&=\Big |\frac{1-\alpha }{M(\alpha )}\Big (\Psi _1(\texttt{t},{\text {x}}(\texttt{t}))-\Psi _1(\texttt{t},{\text {x}}_1(\texttt{t}))\Big ) \\&+\frac{\alpha }{M(\alpha )} \int _{0}^\texttt{t}\Big (\Psi _1(\xi ,{\text {x}}(\xi ))-\Psi _1(\xi ,{\text {x}}_1(\xi ))\Big ) d\xi \Big |\\&\le \frac{1-\alpha }{M(\alpha )} \omega _{1}\left\| {\text {x}}-{\text {x}}_1\right\| +\frac{\alpha }{M(\alpha )} \int _{0}^\texttt{t} \omega _{1}\left\| {\text {x}}-{\text {x}}_1\right\| d\xi \\&\le \Big (\frac{1-\alpha }{M(\alpha )}+\frac{\alpha T}{M(\alpha )} \Big ) \omega _{1}\left\| {\text {x}}-{\text {x}}_1\right\| \text{. } \end{aligned} \end{aligned}$$

Then,

$$\begin{aligned} \Big |{\text {x}}-{\text {x}}_1\Big | \le {\text {v}}_{1} \omega _{1}, \text { with }{\text {v}}_{1}=\Big (\frac{1-\alpha }{M(\alpha )}+\frac{\alpha T}{M(\alpha )} \Big )\left\| {\text {x}}-{\text {x}}_1\right\| . \end{aligned}$$

Similarly, one obtains

$$\begin{aligned} \begin{aligned} \Big |{\text {y}}-{\text {y}}_1\Big |&\le {\text {v}}_{2} \omega _{2}, \text { with } {\text {v}}_{2}=\Big (\frac{1-\alpha }{M(\alpha )}+\frac{\alpha T}{M(\alpha )} \Big )\left\| {\text {y}}-{\text {y}}_1\right\| ,\\ \Big |{\text {z}}-{\text {z}}_1\Big |&\le {\text {v}}_{3} \omega _{3}, \text { with } {\text {v}}_{3}=\Big (\frac{1-\alpha }{M(\alpha )}+\frac{\alpha T}{M(\alpha )} \Big )\left\| {\text {z}}-{\text {z}}_1\right\| . \end{aligned} \end{aligned}$$

Hence, the proof follows.

**Figure 1:**
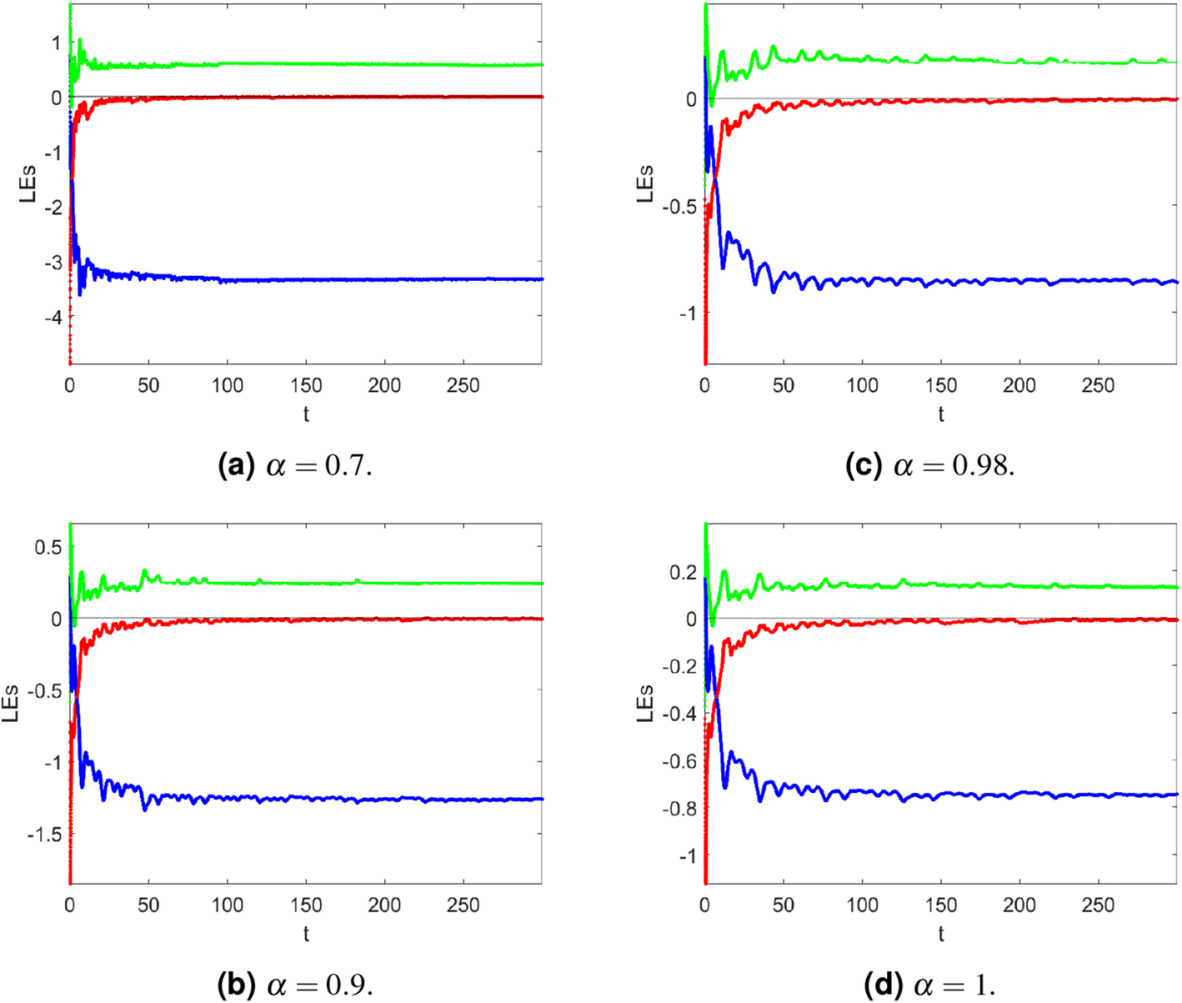
Lyapunov exponent spectrum of a fractional Newton-Leipnik system (2) at $\alpha =0.7, 0.9, 0.98, 1$, receptively.

## Kaplan-Yorke dimension

$$\begin{aligned} \begin{aligned} D_{KY} = j + \frac{\sigma _1+\cdots+\sigma _j}{|\sigma _{j+1}|}. \end{aligned} \end{aligned}$$

where $\sigma _1\le \sigma _n$ are Lyapunov characteristic exponents and *j* is the largest integer for which

$$\begin{aligned} \lambda _1+\cdots +\lambda _j\ge 0. \end{aligned}$$

If $\nu =\sigma =D$, where $\nu$ is the correlation exponent, $\sigma$ the information dimension, and *D* the Hausdorff dimension, then

$$\begin{aligned} D\le D_{KY}. \end{aligned}$$

see^19^.

You can now compute the Lyapunov exponent (LE) of (2) using the Danca algorithm:^5,6^ and apply the Adams-Bashforth-Moulton numerical scheme. Using Table 2, we can see that the Newton-Leipnik system (2) is dissipative, since the sum of the Lyapunov exponents (LE) in each row is negative. Observe that the Lyapunov exponent depends on ${\text {x}}(0)= 0.349$, ${\text {y}}(0)= 0$, and ${\text {z}}(0)= -\,0.160$. Table 1 presents some fractional derivatives with Kaplan-Yorke dimensions:

$$\begin{aligned} \begin{aligned} \dim (LE) = 2 + \frac{LE_1 + LE_2}{ \vert LE_3 \vert }. \end{aligned} \end{aligned}$$

For $$\alpha =0.70$$,

$$\begin{aligned} \dim (LE) = 2 + \frac{0.5745-0.0092}{\vert -3.3237\vert } = 2.17. \end{aligned}$$

For $$\alpha =0.90$$,

$$\begin{aligned} \begin{aligned} \dim (LE) = 2 + \frac{0.3330-0.0082}{\vert -2.0154\vert } =2.161. \end{aligned} \end{aligned}$$

For $$\alpha =0.98$$,

$$\begin{aligned} \begin{aligned} \dim (LE) = 2 + \frac{0.1727-0.0031}{\vert -0.8608\vert } =2.197. \end{aligned} \end{aligned}$$

For $$\alpha =1$$,

$$\begin{aligned} \begin{aligned} \dim (LE) = 2 + \frac{0.1280-0.0085}{\vert -0.7444\vert } =2.160. \end{aligned} \end{aligned}$$

The fact that all of the Kaplan-Yorke dimensions calculated earlier are fractional is another indication that the system is moving in a chaotic direction. Figure 1’s simulation results demonstrate the Lyapunov exponential spectrum technique for chaotic fractional-order systems’ high accuracy and convergence.

When evaluating the Newton-Leipnik dissipative properties, it turns out that the volume element ${\text {y}}_0$ shrinks exponentially to ${\text {y}}_0e^{(-a-0.4+b){\text {t}}}$ at time ${\text {t}}$ and the asymptotic motion eventually becomes an attractor stabilized. The divergent flow of (2) is dissipative if and only if $\nabla V <0,$

$$\begin{aligned} \nabla V = -a - 0.4 + b. \end{aligned}$$

If $b-a< 0.4$ then the system is dissipative.

The system (2) is symmetric about the z axis since it is invariant under the coordinate transformation $({\text {x}}, {\text {y}}, {\text {z}})\rightarrow (-{\text {x}}, -{\text {y}}, -{\text {z}})$. Table 2 displays the Lyapunov exponents for of a fractional Newton-Leipnik system (2). Simulation results in Fig. 1 demonstrate the convergence of the Lyapunov exponential spectrum technique for a fractional Newton-Leipnik system (2).

**Table 2:** Lyapunov exponents versus $\alpha$ of a fractional Newton-Leipnik system (2).

$\alpha$	LE1	LE2	LE3
0.7	0.5745	− 0.0092	− 3.3237
0.9	0.2372	− 0.0073	− 1.2608
0.98	0.1727	− 0.0031	− 0.8608
1	0.1280	− 0.0085	− 0.7444

## Control of Newton-Leipnik systems

The control of the proposed system (2) in sense of Caputo-Fabrizio can be written as:

5 $$\begin{aligned} \begin{aligned} {}^{{\text {CF}}} \mathscr {D}_{{\text {t}}}^{\alpha ({\text {t}})} {\text {x}}({\text {t}})&=-a{\text {x}}({\text {t}})+{\text {y}}({\text {t}})+10{\text {y}}({\text {t}}){\text {z}}({\text {t}})-3.34({\text {x}}({\text {t}}) + {\text {y}}({\text {t}})), \\ {}^{{\text {CF}}} \mathscr {D}_{{\text {t}}}^{\alpha ({\text {t}})} {\text {y}}({\text {t}})&=-{\text {x}}({\text {t}})-0.4{\text {y}}({\text {t}})+5{\text {x}}({\text {t}}){\text {z}}({\text {t}}),\\ {}^{{\text {CF}}} \mathscr {D}_{{\text {t}}}^{\alpha ({\text {t}})}{\text {z}}({\text {t}})&= b{\text {z}}({\text {t}})-5{\text {x}}({\text {t}}){\text {y}}({\text {t}}), \end{aligned} \end{aligned}$$

with $a = 0.4$, $b = 0.175$, $({\text {x}}(0),{\text {y}}(0),{\text {z}}(0))=(0.349, 0,-0.160)$. Evaluating the dissipative properties of Newton-Leipnik, it turns out that the volume element ${\text {y}}_0$ shrinks exponentially at time ${\text {t}}$ in ${\text {y}}_0e^{(-s-1-1){\text {t}}}$, and the asymptotic motion eventually stabilizes as an attractor. Therefore the divergent flow of (5) is dissipative if and only if $\nabla V =\frac{\partial \dot{{\text {x}}}}{\partial u}+\frac{\partial \dot{{\text {y}}}}{\partial v}+\frac{\partial \dot{{\text {z}}}}{\partial w}< 0,$ that is

$$\begin{aligned} \nabla V = -a + k - 0.4 + b. \end{aligned}$$

Thus, if $k-a + b<0.4$, the system is dissipative. By looking at the Lyapunov exponent value, we can see that the fixed point is stable.

For $$\alpha =0.70$$,

$$\begin{aligned} \begin{aligned} \dim (LE) = 2 + \frac{-0.2663-0.2752}{\vert -16.4301\vert } =1.9670. \end{aligned} \end{aligned}$$

For $$\alpha =0.90$$,

$$\begin{aligned} \begin{aligned} \dim (LE) = 2 + \frac{-0.0969-0.1049}{\vert -6.3144 \vert } =1.96804. \end{aligned} \end{aligned}$$

For $$\alpha =0.98$$,

$$\begin{aligned} \begin{aligned} \dim (LE) = 2 + \frac{-0.0658-0.0737}{\vert -4.2426\vert }=1.96411. \end{aligned} \end{aligned}$$

For $$\alpha =1$$,

$$\begin{aligned} \begin{aligned} \dim (LE) = 2 + \frac{-0.0598-0.0677}{\vert -3.8372\vert } =1.9913. \end{aligned} \end{aligned}$$

**Figure 2:**
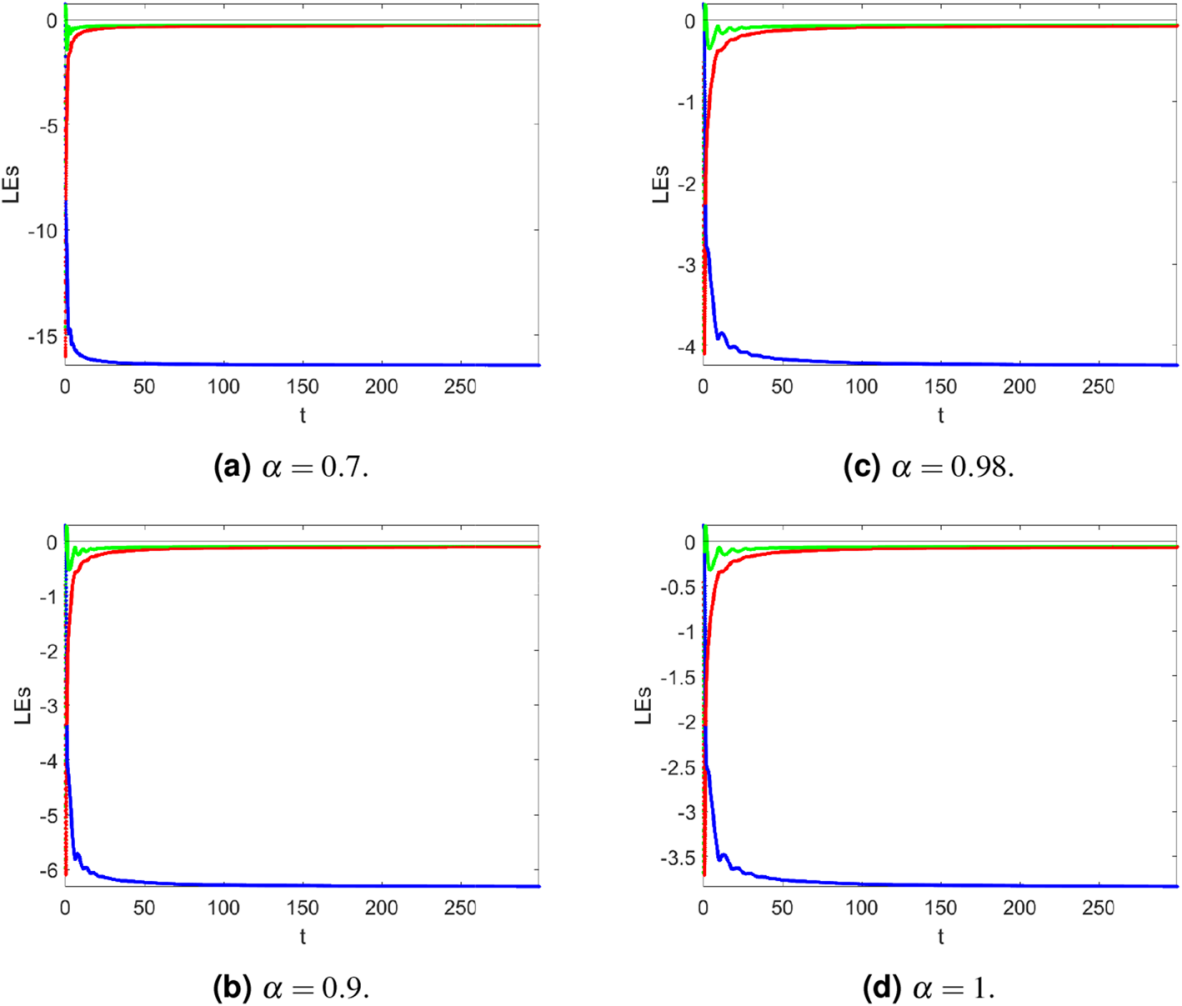
Lyapunov exponent spectrum of a controlled fractional Newton-Leipnik system (5) at $\alpha =0.7, 0.9, 0.98, 1$, receptively.

Table 3 displays the Lyapunov exponents for of a controlled fractional Newton-Leipnik system (5). Simulation results in Figure 2 demonstrate the convergence of the Lyapunov exponential spectrum technique for a fractional Newton-Leipnik system (5).

**Table 3:** Lyapunov exponents versus $\alpha$ of a controlled fractional Newton-Leipnik system (5).

$\alpha$	LE1	LE2	LE3
0.7	− 0.2663	−0.2752	− 16.4301
0.9	− 0.0969	− 0.1049	− 6.3144
0.98	− 0.0658	− 0.0737	− 4.2426
1	− 0.0598	− 0.0677	− 3.8372

## Numerical schemes

### Constant-order numerical schemes in sense of Caputo-Fabrizio

We present the following Cauchy problem with new fractional derivative:

$$\begin{aligned} { }_{0}^{C F} D_{t}^{\alpha } u(t)=h(t, u(t)). \end{aligned}$$

From the definition of the Caputo-Fabrizio integral, we can reformulate the above equation as

6 $$\begin{aligned} u(t)-u(0)=\frac{1-\alpha }{M(\alpha )} h(t, u(t))+\frac{\alpha }{M(\alpha )} \int _{0}^{t} h(\tau , u(\tau )) d \tau . \end{aligned}$$

We write Eq. (6) at the point $t_{k+1}=(k+1) \Delta t$,

$$\begin{aligned} u\left( t_{k+1}\right) -u(0)=\frac{1-\alpha }{M(\alpha )} h\left( t_{k}, u\left( t_{k}\right) \right) +\frac{\alpha }{M(\alpha )} \int _{0}^{t_{k+1}} h(\tau , u(\tau )) d \tau \end{aligned}$$

and at the point $t_{k}=k \Gamma t$,

$$\begin{aligned} u\left( t_{k}\right) -u(0)=\frac{1-\alpha }{M(\alpha )} h\left( t_{k-1}, u\left( t_{k-1}\right) \right) +\frac{\alpha }{M(\alpha )} \int _{0}^{t_{k}} h(\tau , u(\tau )) d \tau . \end{aligned}$$

Taking the difference of these equations, we can write the following:

$$\begin{aligned} \begin{aligned} u\left( t_{k+1}\right) -u\left( t_{k}\right)&=\frac{1-\alpha }{M(\alpha )}\left[ h\left( t_{k}, u\left( t_{k}\right) \right) -h\left( t_{k-1}, u\left( t_{k-1}\right) \right) \right] \\&\quad +\frac{\alpha }{M(\alpha )} \int _{t_{k}}^{t_{k+1}} h(\tau , u(\tau )) d \tau . \end{aligned} \end{aligned}$$

Putting its Lagrange polynomial into the above equation, we can get the following:

$$\begin{aligned} \begin{aligned} u^{k+1}-u^{k}&=\frac{1-\alpha }{M(\alpha )}\left[ h\left( t_{k}, u\left( t_{k}\right) \right) -h\left( t_{k-1}, u\left( t_{k-1}\right) \right) \right] \\&\quad+\frac{\alpha }{M(\alpha )} \int _{t_{k}}^{t_{k+1}}\left\{ \begin{array}{c} \frac{h\left( t_{k}, u\left( t_{k}\right) \right) }{\Gamma t}\left( \tau -t_{k-1}\right) -\frac{h\left( t_{k-1}, u\left( t_{k-1}\right) \right) }{\Gamma t}\left( \tau -t_{k}\right) \end{array}\right\} d \tau , \end{aligned} \end{aligned}$$

and we get the following:

$$\begin{aligned} \begin{aligned} u^{k+1}-u^{k}&=\frac{1-\alpha }{M(\alpha )}\left[ h\left( t_{k}, u\left( t_{k}\right) \right) -h\left( t_{k-1}, u\left( t_{k-1}\right) \right) \right] \\&\quad+\frac{\alpha }{M(\alpha )}\left\{ \begin{array}{c} \frac{h\left( t_{k}, u\left( t_{k}\right) \right) }{\Gamma t} \int _{t_{k}}^{t_{k+1}}\left( \tau -t_{k-1}\right) d \tau -\frac{h\left( t_{k-1}, u\left( t_{k-1}\right) \right) }{\Gamma t} \int _{t_{k}}^{t_{k+1}}\left( \tau -t_{k}\right) d \tau \end{array}\right\} . \end{aligned} \end{aligned}$$

The integrals on the right hand side of the above equation can be calculated as

$$\begin{aligned} \begin{aligned} \int _{t_{k}}^{t_{k+1}}\left( \tau -t_{k-1}\right) d \tau&=\frac{3}{2}(\Gamma t)^{2}, \\ \int _{t_{k}}^{t_{k+1}}\left( \tau -t_{k}\right) d \tau&=\frac{1}{2}(\Gamma t)^{2}. \end{aligned} \end{aligned}$$

Thus, we have the following numerical scheme:

$$\begin{aligned} \begin{aligned} u^{k+1}&=u^{k}+\frac{1-\alpha }{M(\alpha )}\left[ h\left( t_{k}, u\left( t_{k}\right) \right) -h\left( t_{k-1}, u\left( t_{k-1}\right) \right) \right] \\ {}&+\frac{\alpha }{M(\alpha )}\left\{ h\left( t_{k}, u^{k}\right) \frac{3}{2} \Gamma t-h\left( t_{k-1}, u^{k-1}\right) \frac{1}{2} \Gamma t\right\} . \end{aligned} \end{aligned}$$

### Variable-order numerical schemes in sense of Caputo-Fabrizio

The following variable-order numerical schemes is the same as^22,28^. A variable order fractional differential equation is shown below.

$$\begin{aligned} { }_{0}^{CF} \mathscr {D}_{{\text {t}}}^{\alpha ({\text {t}})} {\text {x}}({\text {t}})={\text {f}}({\text {t}}, {\text {x}}({\text {t}})) \text{. } \end{aligned}$$

The fundamental theorem of fractions has been applied, and we have

$$\begin{aligned} {\text {x}}({\text {t}})-{\text {x}}(0)=\frac{1-\alpha ({\text {t}})}{\Lambda (\alpha ({\text {t}}))} {\text {f}}({\text {t}}, {\text {x}}({\text {t}}))+\frac{\alpha ({\text {t}})}{\Lambda (\alpha ({\text {t}}))} \int _{0}^{{\text {t}}} {\text {f}}(\theta , {\text {x}}(\theta )) d \theta . \end{aligned}$$

In this way

7 $$\begin{aligned} \begin{aligned} {\text {x}}({\text {t}}_{n+1})-{\text {x}}(0)&= \frac{(2-\alpha ({\text {t}}))(1-\alpha ({\text {t}}))}{2} {\text {f}}({\text {t}}_{n}, {\text {x}}({\text {t}}_{n})) +\frac{\alpha ({\text {t}})(2-\alpha ({\text {t}}))}{2} \int _{0}^{{\text {t}}_{n+1}} {\text {f}}({\text {t}}, {\text {x}}({\text {t}})) d{\text {t}}, \end{aligned} \end{aligned}$$

and

8 $$\begin{aligned} \begin{aligned} {\text {x}}({\text {t}}_{n})-{\text {x}}(0)=&\frac{(2-\alpha ({\text {t}}))(1-\alpha ({\text {t}}))}{2} {\text {f}}({\text {t}}_{n-1}, {\text {x}}({\text {t}}_{n-1}))+\frac{\alpha ({\text {t}})(2-\alpha ({\text {t}}))}{2} \int _{0}^{{\text {t}}_{n}} {\text {f}}({\text {t}}, {\text {x}}({\text {t}})) d{\text {t}}. \end{aligned} \end{aligned}$$

When (7) is substituted for (8), we get

$$\begin{aligned} \begin{aligned} {\text {x}}({\text {t}}_{n+1})&= {\text {x}}({\text {t}}_{n})+\frac{(2-\alpha ({\text {t}}))(1-\alpha ({\text {t}}))}{2}\times [{\text {f}}({\text {t}}_{n}, {\text {x}}({\text {t}}_{n}))-{\text {f}}({\text {t}}_{n-1}, {\text {x}}({\text {t}}_{n-1}))] \\&\quad+\frac{\alpha ({\text {t}})(2-\alpha ({\text {t}}))}{2} \int _{{\text {t}}_{n}}^{{\text {t}}_{n+1}} {\text {f}}({\text {t}}, {\text {x}}({\text {t}})) d{\text {t}}, \end{aligned} \end{aligned}$$

where

$$\begin{aligned} \int _{{\text {t}}_{n}}^{{\text {t}}_{n+1}} {\text {f}}({\text {t}}, {\text {x}}({\text {t}})) d{\text {t}}=\frac{3h}{2} {\text {f}}({\text {t}}_{n}, {\text {x}}_{n})-\frac{h}{2} {\text {f}}({\text {t}}_{n-1}, {\text {x}}_{n-1}). \end{aligned}$$

The numerical solution is given by

$$\begin{aligned} \begin{aligned} {\text {x}}_{n+1}&= {\text {x}}_{n}+\Big [\frac{(2-\alpha ({\text {t}}))(1-\alpha ({\text {t}}))}{2}+\frac{3 h}{4} \alpha ({\text {t}})(2-\alpha ({\text {t}}))\Big ] {\text {f}}({\text {t}}_{n}, {\text {x}}_{n}) \\&\quad-\Big [\frac{(2-\alpha ({\text {t}}))(1-\alpha ({\text {t}}))}{2}+\frac{h}{4} \alpha ({\text {t}})(2-\alpha ({\text {t}}))\Big ] {\text {f}}({\text {t}}_{n-1}, {\text {x}}_{n-1}). \end{aligned} \end{aligned}$$

The system (2) is given by

$$\begin{aligned} \begin{aligned} {\text {x}}_{n+1}({\text {t}})&= {\text {x}}_{n}+\Big [\frac{(2-\alpha ({\text {t}}))(1-\alpha ({\text {t}}))}{2}+\frac{3 h}{4} \alpha ({\text {t}})(2-\alpha ({\text {t}}))\Big ] \times \Psi _1({\text {t}}_{n}, {\text {x}}_{n}({\text {t}}), {\text {y}}_{n}({\text {t}}), {\text {z}}_{n}({\text {t}})) \\&\quad-\Big [\frac{(2-\alpha ({\text {t}}))(1-\alpha ({\text {t}}))}{2}+\frac{h}{4} \alpha ({\text {t}})(2-\alpha ({\text {t}}))\Big ] \times \Psi _1({\text {t}}_{n-1}, {\text {x}}_{n-1}({\text {t}}), {\text {y}}_{n-1}({\text {t}}), {\text {z}}_{n-1}({\text {t}})), \\ {\text {y}}_{n+1}({\text {t}})&= {\text {y}}_{n}+\Big [\frac{(2-\alpha ({\text {t}}))(1-\alpha ({\text {t}}))}{2}+\frac{3 h}{4} \alpha ({\text {t}})(2-\alpha ({\text {t}}))\Big ] \times \Psi _2({\text {t}}_{n}, {\text {x}}_{n}({\text {t}}), {\text {y}}_{n}({\text {t}}), {\text {z}}_{n}({\text {t}}))\\&\quad -\Big [\frac{(2-\alpha ({\text {t}}))(1-\alpha ({\text {t}}))}{2}+\frac{h}{4} \alpha ({\text {t}})(2-\alpha ({\text {t}}))\Big ] \times \Psi _2({\text {t}}_{n-1}, {\text {x}}_{n-1}({\text {t}}), {\text {y}}_{n-1}({\text {t}}), {\text {z}}_{n-1}({\text {t}})), \\ {\text {z}}_{n+1}({\text {t}})&= {\text {z}}_{n}+\Big [\frac{(2-\alpha ({\text {t}}))(1-\alpha ({\text {t}}))}{2}+\frac{3 h}{4} \alpha ({\text {t}})(2-\alpha ({\text {t}}))\Big ] \times \Psi _3({\text {t}}_{n}, {\text {x}}_{n}({\text {t}}), {\text {y}}_{n}({\text {t}}), {\text {z}}_{n}({\text {t}})) \\&\quad -\Big [\frac{(2-\alpha ({\text {t}}))(1-\alpha ({\text {t}}))}{2}+\frac{h}{4} \alpha ({\text {t}})(2-\alpha ({\text {t}}))\Big ] \times \Psi _3({\text {t}}_{n-1}, {\text {x}}_{n-1}({\text {t}}), {\text {y}}_{n-1}({\text {t}}), {\text {z}}_{n-1}({\text {t}})), \end{aligned} \end{aligned}$$

where $\Psi _1$, $\Psi _2$, $\Psi _3$ are defined as in section “Model analysis”.

**Figure 3:**
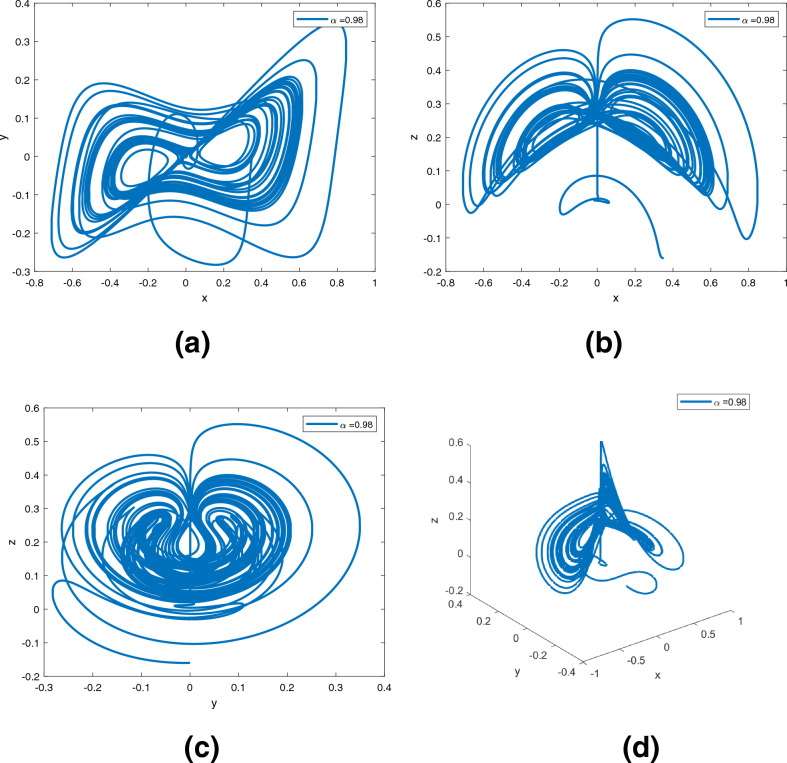
Dynamics of the system (2) in $({\text {x}}, {\text {y}}), ({\text {x}}, {\text {z}}), ({\text {y}}, {\text {z}}), ({\text {x}}, {\text {y}}, {\text {z}})$ planes with $\alpha ({\text {t}})=0.98$, respectively in (**a**)–(**d**).

**Figure 4:**
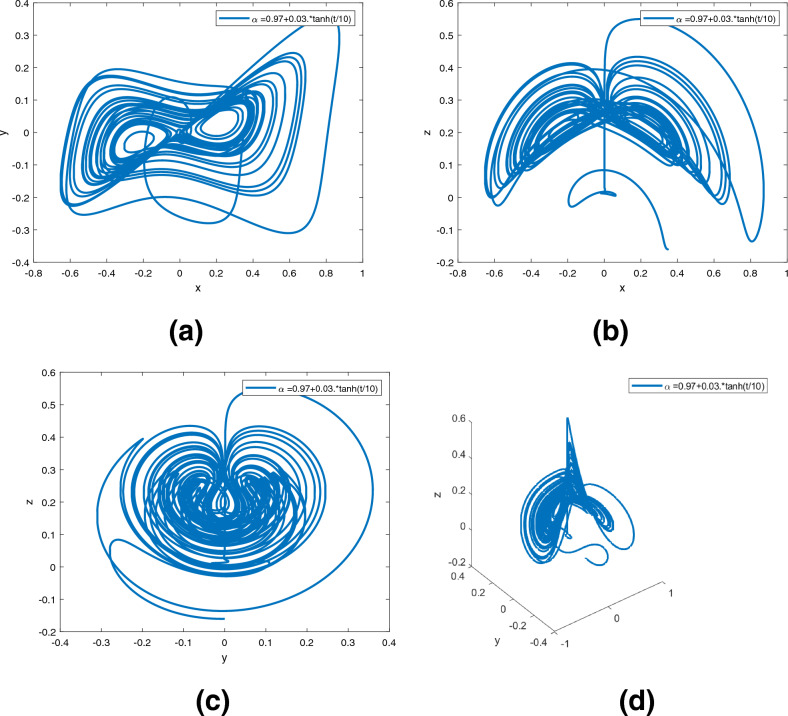
Dynamics of the system (2) in $({\text {x}}, {\text {y}}), ({\text {x}}, {\text {z}}), ({\text {y}}, {\text {z}}), ({\text {x}}, {\text {y}}, {\text {z}})$ planes with $\alpha ({\text {t}})=0.97+0.03 \text { tansh }(t/10)$, respectively in (**a**)–(**d**).

**Figure 5:**
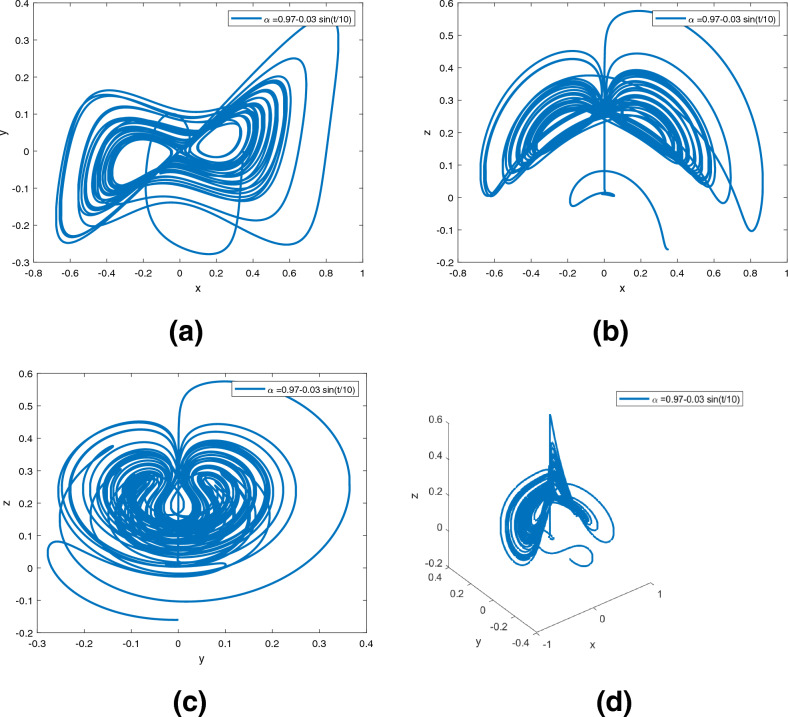
Dynamics of the system (2) in $({\text {x}}, {\text {y}}), ({\text {x}}, {\text {z}}), ({\text {y}}, {\text {z}}), ({\text {x}}, {\text {y}}, {\text {z}})$ planes with $\alpha ({\text {t}})=0.97-0.03\text {sin }(t/10)$, respectively in (**a**)–(**d**).

**Figure 6:**
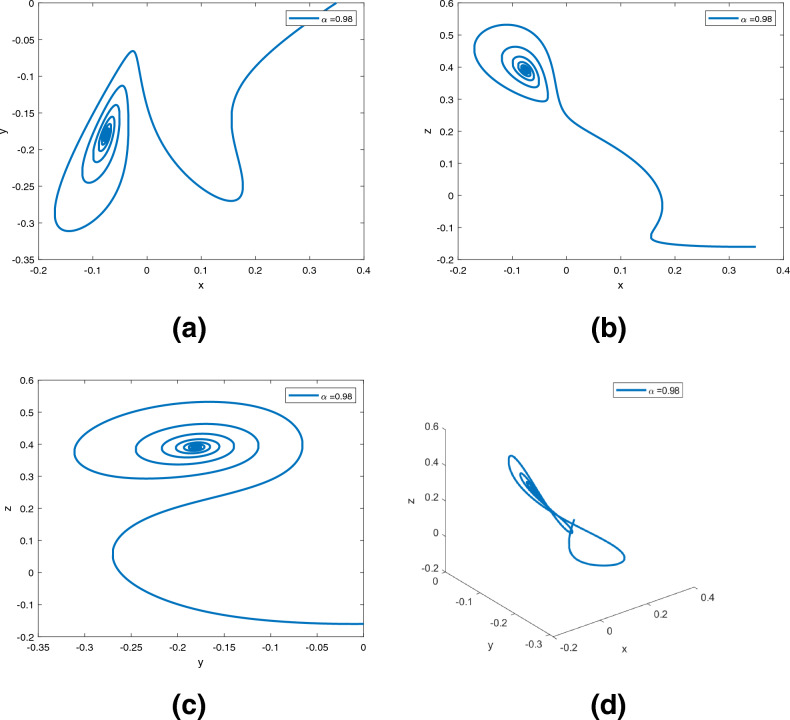
Dynamics of the controlled system (5) in $({\text {x}}, {\text {y}}), ({\text {x}}, {\text {z}}), ({\text {y}}, {\text {z}}), ({\text {x}}, {\text {y}}, {\text {z}})$ planes with $\alpha ({\text {t}})=0.98$, respectively in (**a**)–(**d**).

**Figure 7:**
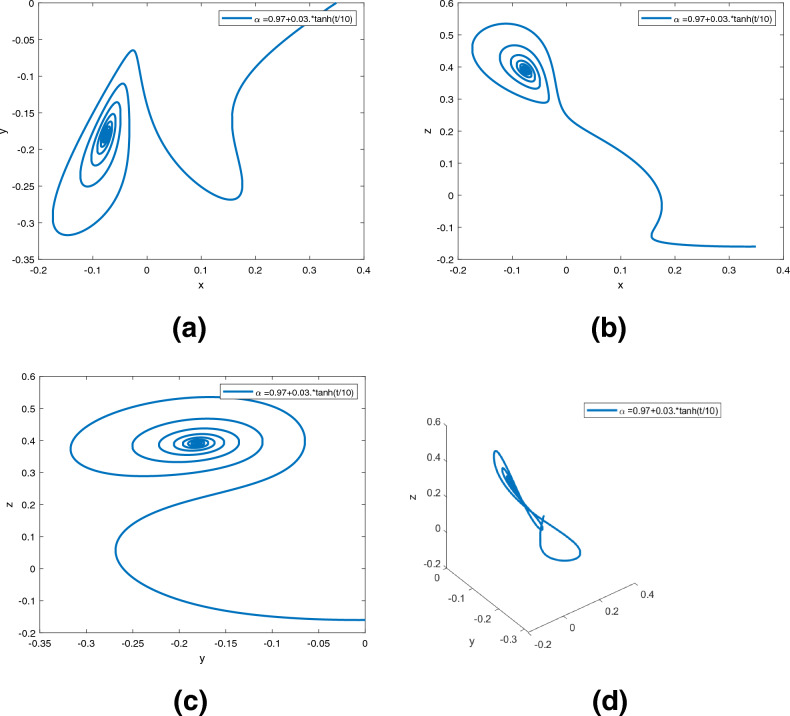
Dynamics of the controlled system (5) in $({\text {x}}, {\text {y}}), ({\text {x}}, {\text {z}}), ({\text {y}}, {\text {z}}), ({\text {x}}, {\text {y}}, {\text {z}})$ planes with $\alpha ({\text {t}})=0.97+0.03\text {tansh }(t/10)$, respectively in (**a**)–(**d**).

**Figure 8:**
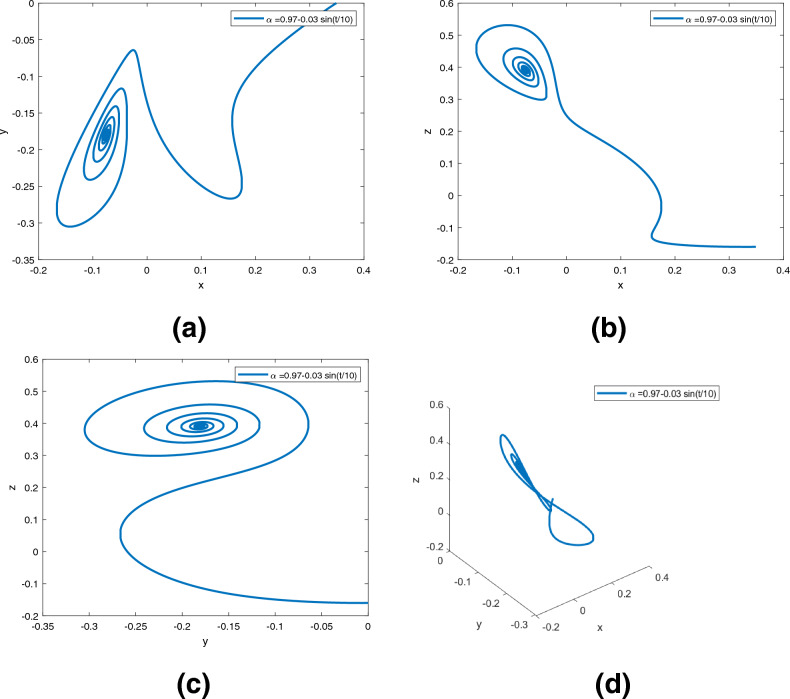
Dynamics of the controlled system (5) in $({\text {x}}, {\text {y}}), ({\text {x}}, {\text {z}}), ({\text {y}}, {\text {z}}), ({\text {x}}, {\text {y}}, {\text {z}})$ planes with $\alpha ({\text {t}})=0.97-0.03\text { sin }(t/10)$, respectively in (**a**)–(**d**).

**Figure 9:**
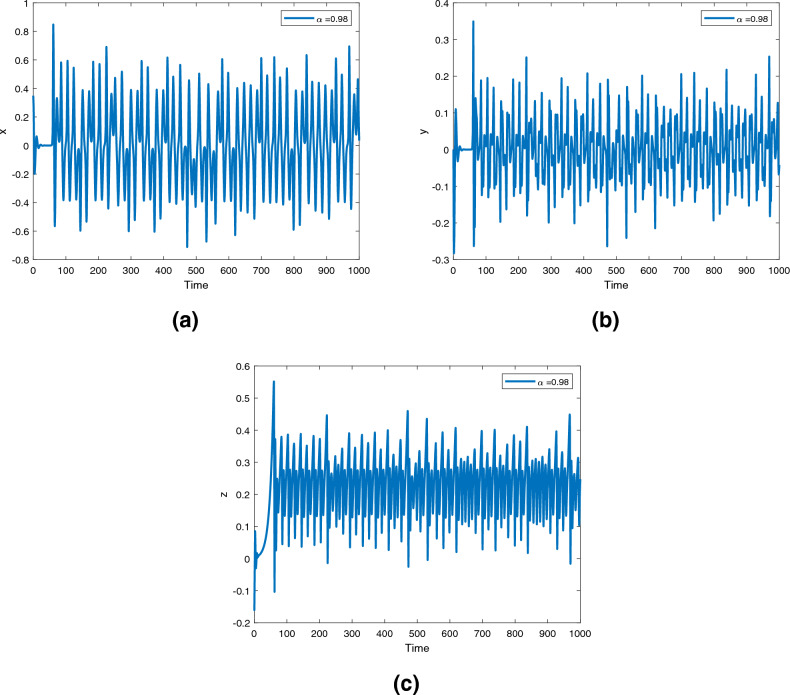
Time series for the fractional order Newton-Leipnik system (2) with fractional order $\alpha ({\text {t}})=0.98$.

**Figure 10:**
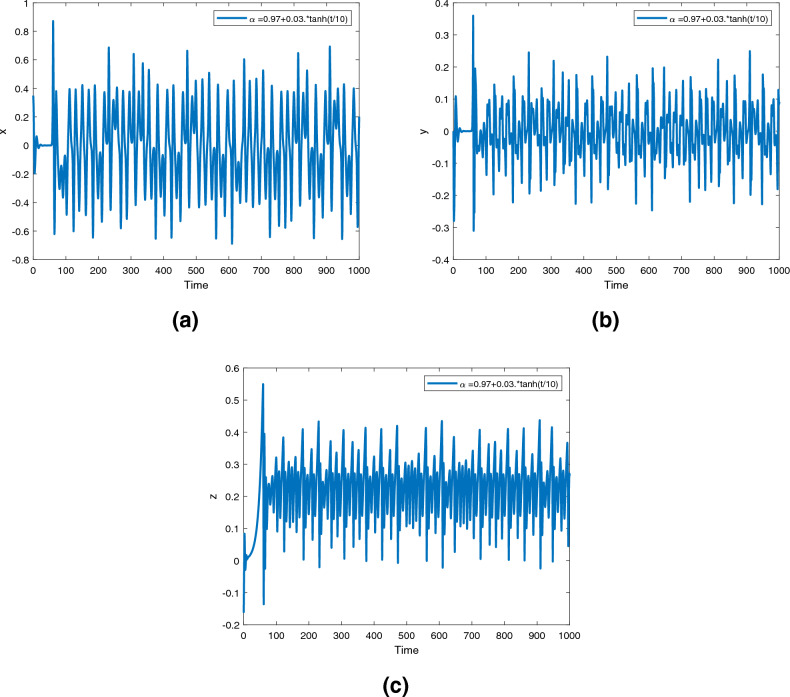
Time series for the fractional order Newton-Leipnik system (2) with fractional order $\alpha ({\text {t}})=0.97+0.03 \text {tansh }(t/10)$.

**Figure 11:**
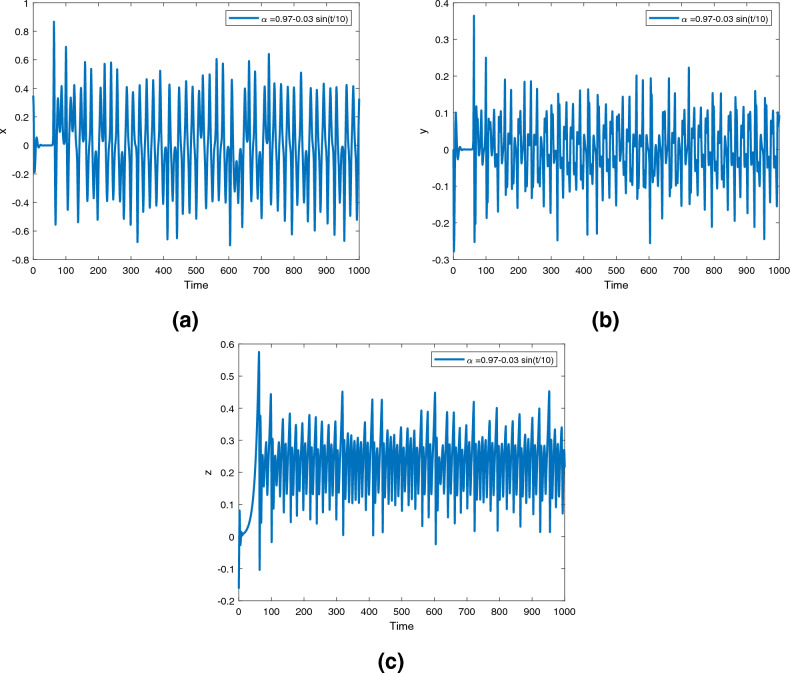
Time series for the fractional order Newton-Leipnik system (2) with fractional order $\alpha ({\text {t}})=0.97-0.03 \text { sin }(t/10)$.

**Figure 12:**
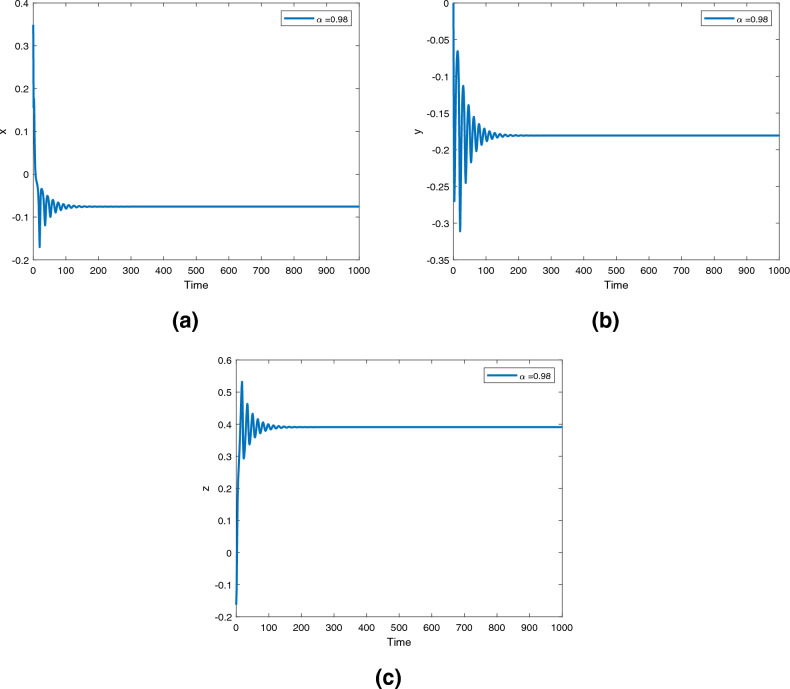
Time series for the controlled fractional order Newton-Leipnik system (5) with fractional order $\alpha ({\text {t}})=0.98$.

**Figure 13:**
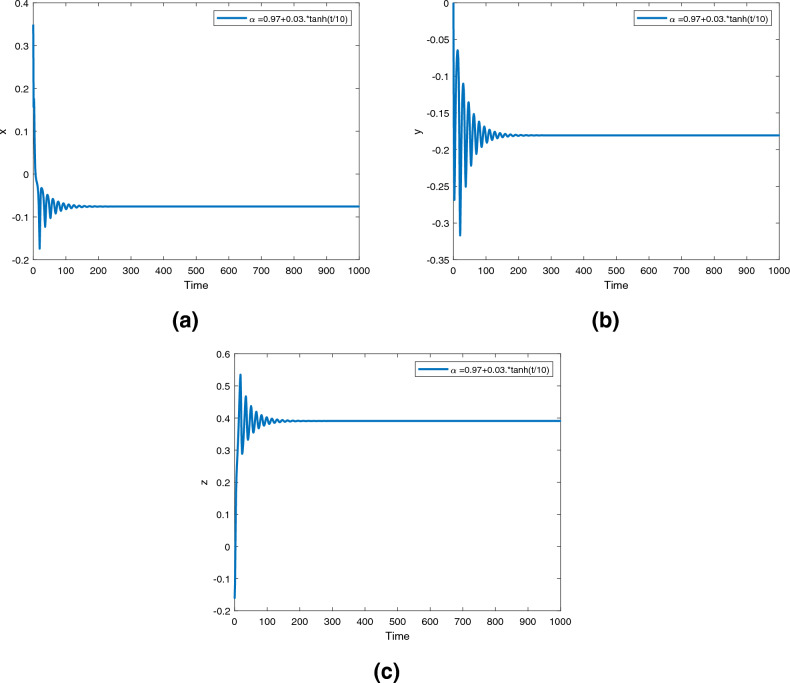
Time series for the controlled fractional order Newton-Leipnik system (5) with fractional order $\alpha ({\text {t}})=0.97+0.03 \text { tansh }(t/10)$.

**Figure 14:**
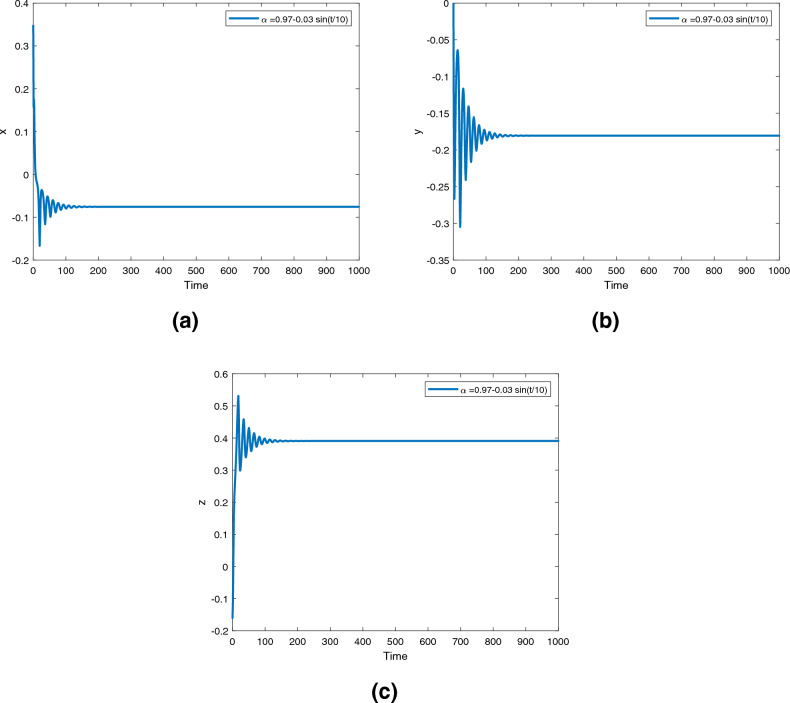
Time series for the controlled fractional order Newton-Leipnik system (5) with fractional order $\alpha ({\text {t}})=0.97-0.03 \text { sin }(t/10)$.

### Numerical simulation

Figures 3, 4, 5 are phase trajectories of system (2) projected onto $({\text {x}}, {\text {y}}), ({\text {x}}, {\text {z}}), ({\text {y}}, {\text {z}}), ({\text {x}}, {\text {y}}, {\text {z}})$ for derivative order $\alpha ({\text {t}})=0.98, 0.97+0.03\text { tansh }(t/10), 0.97-0.03 \text { sin }(t/10)$ with parameters $a = 0.4, b = 0.175$. The simulation time is 1000s, the time step h = 0.01, with $({\text {x}}(0), {\text {y}}(0), {\text {z}}(0)) = (0.349,0.0,-0.160)$. We can observe that double scroll attractor surrounded the equilibria $E_2$ and $E_5$.

Figures 6, 7 and 8 are phase trajectories of system (5) projected onto $({\text {x}}, {\text {y}}), ({\text {x}}, {\text {z}}), ({\text {y}}, {\text {z}}), ({\text {x}}, {\text {y}}, {\text {z}})$ for derivative order $\alpha ({\text {t}})= 0.98, 0.97+0.03\text { tansh }(t/10), 0.97-0.03 \text { sin }(t/10)$ with parameters $a = 0.4, b = 0.175$. The simulation time is 1000s, the time step h = 0.01, and the initial conditions are: $({\text {x}}(0), {\text {y}}(0), {\text {z}}(0)) = (0.349,0.0,-0.160)$.

Figures 9, 10 and 11 are phase trajectories of the time series of $({\text {x}}, {\text {y}}), ({\text {x}}, {\text {z}}), ({\text {y}}, {\text {z}}), ({\text {x}}, {\text {y}}, {\text {z}})$ for system (2) with fractional order $\alpha ({\text {t}})=1, 0.98, 0.97+0.03\text { tansh }(t/10), 0.97-0.03 \text { sin }(t/10)$ with parameters $a = 0.4, b = 0.175$.

Figures 12, 13 and 14 are phase trajectories of the time series of $({\text {x}}, {\text {y}}), ({\text {x}}, {\text {z}}), ({\text {y}}, {\text {z}}), ({\text {x}}, {\text {y}}, {\text {z}})$ for system (5) with fractional order $\alpha ({\text {t}})=1, 0.98, 0.97+0.03\text { tansh }(t/10), 0.97-0.03 \text { sin }(t/10)$ with parameters $a = 0.4, b = 0.175$.

## Discussion of results

A fractional mathematical model based on the Caputo-Fabrizio operator was developed to describe the Newton-Leipnik system (2). Furthermore, the results depicted in Figs. 3, 4,5, 6, 7, 8, 9, 10, 12 and 13, with the decreasing and increasing values of $\alpha$, can observe how effective infection is in the model’s behavior. The equilibrium points of the system (2) and the corresponding eigenvalues of the Jacobian matrix are shown in Table 1. In chaotic 3D chaos, the equilibrium points of the Newton-Leipnik system (2) yield all unstable eigenvalues as illustrated in Table 1. A balance with exactly five unstable eigenvalues, the saddle point or saddle focus with index 2, is responsible for the generation of the rolling attractor. Therefore, the theoretically calculated minimum effective size of the Newton-Leipnik system is 2.82 as illustrated in Sheu et al.^4^, and this finding is further verified in the numerical simulation results in section “Control of Newton-Leipnik systems”. The system shows good dynamic behavior.

## Conclusions

A fractional mathematical model based on Caputo-Fabrizio fractional operators is presented. Thus, in this paper, we examined the dynamics of the Newton-Leipnik system under the fractional derivative with non-singular kernels. Via the fixed-point theorems of Sachuder and Banach, we have explained the existence theory of the model under the Caputo-Fabrizio fractional operator. We have used the fixed point approach for the existence of a unique solution under the non-singular operator. An approximate solution to the model has been calculated using the Caputo-Fabrizio fractional scheme. The results show that the system is stable at equilibrium points and each obtained function converges at its equilibrium point. To investigate the effect of derivative order on the model results, the functions obtained from the model are plotted for different fraction degrees. The results show that the general behavior of the functions is the same with small changes in derivative order but the numerical results are different. The model can also be controlled linearly. We have noticed that the nonsingular fractional operator produces excellent results for the model under consideration compared to the Caputo-Fabrizio and fractional operators. Thus, we have concluded that modeling with the nonsingular operator is much better than modeling with the singular operator.

## Data Availability

The authors confirm that the data supporting the findings of this study are available within the article.

## References

[1] Wang, X. & Tian, L. Bifurcation analysis and linear control of the Newton-Leipnik system. *Chaos Soliton. Fract.* **27**, 31–8 (2006).

[2] Chen, H. K. & Lin, T. N. Synchronization of chaotic symmetric gyros by one-way coupling conditions. *ImechE J. Mech. Eng. Sci.* **217**, 331–40 (2003).

[3] Chen, H. K. & Lee, C. I. Anti-control of chaos in rigid body motion. *Chaos Soliton. Fract.* **21**, 957–65 (2004).

[4] Sheu, L. J. *et al.* Chaos in the Newton-Leipnik system with fractional order. *Chaos Soliton. Fract.* **36**, 98–103 (2008).

[5] Danca, M. F. Lyapunov exponents of a discontinuous 4D hyperchaotic system of integer or fractional order. *Entropy* **20**(5), 337 (2018). 33265427 10.3390/e20050337 PMC7512856

[6] Danca, M. F. & Kuznetsov, N. Matlab code for Lyapunov exponents of fractional-order systems. *Int. J. Bif. Chaos* **28**(5), 1850067 (2018).

[7] Deng, W., Li, C. & Lu, J. Stability analysis of linear fractional differential system with multiple time delays. *Nonlinear Dyn.* **48**, 409–416 (2007).

[8] Ge, Z. M. & Chen, H. K. Stability and chaotic motions of a symmetric heavy gyroscope. *Jpn. J. Appl. Phys.* **35**, 1954–65 (1996).

[9] Leipnik, R. B. & Newton, T. A. Double strange attractors in rigid body motion. *Phys. Lett. A* **86**, 63–7 (1981).

[10] Sheu, L.-J. *et al.* Chaos in the Newton-Leipnik system with fractional order. *Chaos Soliton. Fract.* **36**, 98–103 (2008).

[11] Ge, Z. M., Chen, H. K. & Chen, H. H. The regular and chaotic Motions of a symmetric heavy gyroscope with harmonic excitation. *J. Sound Vibr.* **198**, 131–47 (1996).

[12] Richter, H. Controlling chaotic system with multiple strange attractors. *Phys. Lett. A* **300**, 182–8 (2002).

[13] Sun, H. H., Abdelwahed, A. A. & Onaral, B. Linear approximation for transfer function with a pole of fractional order. *IEEE Trans. Autom. Control* **29**, 441–4 (1984).

[14] Podlubny, I. *Fractional Differential Equations* (Academic Press, 1999).

[15] Losada, J. & Nieto, J. J. Properties of a new fractional derivative without singular kernel. *Prog. Fract. Differ. Appl.* **1**(2), 87–92 (2015).

[16] Atangana, A. & Gomez-Aguilar, J. F. Fractional derivatives with no-index law property: Application to chaos and statistics. *Chaos Soliton. Fract.* **114**, 516–535 (2018).

[17] Atangana, A. S. Q. Modeling attractors of chaotic dynamical systems with fractal-fractional operators. *Chaos Soliton. Fract.* **123**, 320–337 (2019).

[18] Atangana, A., Akgal, A. & Owolabi, K. M. Analysis of fractal fractional differential equations. *Alexandr. Eng. J.* **1**, 1–12 (2020).

[19] Grassberger, P. & Procaccia, I. Measuring the strangeness of strange attractors. *Phys. D* **9**, 189–208 (1983).

[20] Hunter, J. K. & Nachtergaele, B. *Applied Analysis* (World Scientific, 2001).

[21] Kreyszig, E. *Introductory Functional Analysis with Applications* (Wiley, 1978).

[22] Abdon, A. & Seda, A. *New Numerical Scheme With Newton Polynomial Theory, Methods, and Applications, Mara Conner Editorial Project Manager: Aleksandra Packowska Production Project Manager: Bharatwaj Varatharajan Designer: Matthew Limbert* (2023).

[23] Bagley, R. L. & Calico, R. A. Fractional order state equations for the control of viscoelastically damped structures. *J. Guid. Control Dyn.* **14**, 304–11 (1991).

[24] Chen, H. K. Chaos and chaos synchronization of a symmetric gyro with linear-plus-cubic damping. *J. Sound Vibr.* **255**, 719–40 (2002).

[25] Tavazoei, M. S. & Haeri, M. A necessary condition for double scroll attractor existence in fractional order systems. *Phys. Lett. A* **367**, 102–113 (2007).

[26] Tavazoei, M. S. & Haeri, M. Chaotic attractors in incommensurate fractional order systems. *Phys. D* **237**, 2628–2637 (2008).

[27] Tong, X. & Mrad, N. Chaotic motion of a symmetric gyro subjected to a harmonic base excitation. *Trans. ASME J. Appl. Mech.* **68**, 681–4 (2001).

[28] Toufik, M. & Atangana, A. New numerical approximation of fractional derivative with non-local and non-singular kernel: Application to chaotic models. *Eur. Phys. J. Plus* **132**(10), 1–16 (2017).

[29] Ulam, S. M. *A Collection of Mathematical Problems* (Interscience, 1960).

[30] Ulam, S. M. *Problems in Modern Mathematics*. The authors oversight the many recently published papers on C-F, fractional operator, fractal-fractional, chaos etc. The introduction and the reference section should improve by considering the following closely related papers (Dover Publications, 2004).

[31] Farman, M., Besbes, H., Nisar, K. S. & Omri, M. Analysis and dynamical transmission of Covid-19 model by using Caputo-Fabrizio derivative. *Alexandr. Eng. J.* **66**, 597–606. 10.1016/j.aej.2022.12.026 (2023).

[32] Ali, A. K. *et al.* Effects of carbon nanotubes on magnetohydrodynamic flow of methanol based nanofluids via Atangana-Baleanu and Caputo-Fabrizio fractional derivatives. *Therm. Sci.* **23**, 883–898. 10.2298/TSCI180116165A (2019).

[33] Shaikh, A. *et al.* Analysis of differential equations involving Caputo-Fabrizio fractional operator and its applications to reaction-diffusion equations. *Adv. Differ. Equ.* **2019**, 178. 10.1186/s13662-019-2115-3 (2019).

[34] Nisar, K. S., Farman, M., Hincal, E. & Shehzad, A. Modelling and analysis of bad impact of smoking in society with Constant Proportional-Caputo Fabrizio operator. *Chaos Soliton. Fract.* **172**, 113549. 10.1016/j.chaos.2023.113549 (2023).

[35] Nisar, K. S., Farman, M., Abdel-Aty, M. & Cao, J. A review on epidemic models in sight of fractional calculus. *Alexandr. Eng. J.* **75**, 81–113. 10.1016/j.aej.2023.05.071 (2023).

[36] Nisar, K. S., Farman, M., Abdel-Aty, M. & Cao, J. Mathematical epidemiology: A review of the singular and non-singular Kernels and their applications. *Progr. Fract. Differ. Appl.* **9**(4), 507–544. 10.18576/pfda/090401 (2023).

[37] M. H. Alshehri, F. Z. Duraihem & S. Saber, Dynamical analysis of fractional-order of IVGTT glucose–insulin interaction, *International Journal of Nonlinear Sciences and Numerical Simulation,* **24**(3) 1123–1140. 10.1515/ijnsns-2020-0201 (2023).

[38] Saber, S. Alghamdi, A. M. Ahmed, G. A. & Alshehri, K. M. Mathematical modelling and optimal control of pneumonia disease in sheep and goats in Al-Baha region with cost-effective strategies. *AIMS Mathematics*, **7** 12011–12049. 10.3934/math.2022669 (2022).

[39] Alalyani, A. & Saber, S. Stability analysis and numerical simulations of the fractional COVID-19 pandemic model, *Int. J. Nonlin. Sci. Num.*, **2022,** 1–14. 10.1515/ijnsns-2021-0042 (2022).

[40] Saber, S. & Alalyani, A. Stability analysis and numerical simulations of IVGTT glucose-insulin interaction models with two time delays. *Mathematical Modelling and Analysis*, **27**(3), 383–407. 10.3846/mma.2022.14007 (2022).

[41] Al-Zahrani, S. M., Elsmih, F. E. I., Al-Zahrani, K. S. & Saber, S., A Fractional Order SITR Model for Forecasting of Transmission of COVID-19: Sensitivity Statistical Analysis, Malaysian Journal of Mathematical Sciences **16**(3), 517–536 (2022).

[42] Sayed Saber, Azza M. Alghamdi, Ghada A. Ahmed & Khulud M. Alshehri. Mathematical Modelling and optimal control of pneumonia disease in sheep and goats in Al-Baha region with cost-effective strategies[J]. *AIMS Mathematics*. **7**(7), 12011–12049. 10.3934/math.2022669 (2022).

[43] Alshehri, Mansoor H., Saber, Sayed & Duraihem Faisal Z. Dynamical analysis of fractional-order of IVGTT glucose–insulin interaction.* International Journal of Nonlinear Sciences and Numerical Simulation*, ** 24,**(3), 1123–1140. 10.1515/ijnsns-2020-0201 (2023).

[44] Alalyani, Ahmad & Saber, Sayed. Stability analysis and numerical simulations of the fractional COVID-19 pandemic model. *International Journal of Nonlinear Sciences and Numerical Simulation,* vol. **24**(3), 989–1002. 10.1515/ijnsns-2021-0042 (2023).

[45] Khalid I.A. Ahmed, Haroon D.S. Adam, M.Y. Youssif & Sayed Saber, Different strategies for diabetes by mathematical modeling: Modified Minimal Model, *Alexandria Engineering Journal,* Volume **80,** 74–87. 10.1016/j.aej.2023.07.050 (2023).

[46] Khalid I.A. Ahmed, Haroon D.S. Adam, M.Y. Youssif & Sayed Saber, Different strategies for diabetes by mathematical modeling: Applications of fractal–fractional derivatives in the sense of Atangana–Baleanu, *Results in Physics,* Volume **52,** 106892. 10.1016/j.rinp.2023.106892 (2023).

[47] Najat Almutairi, Sayed Saber, Hijaz Ahmad. The fractal-fractional Atangana-Baleanu operator for pneumonia disease: stability, statistical and numerical analyses[J]. *AIMS Mathematics*, **8**(12), 29382–29410. 10.3934/math.20231504 (2023).

[48] Sayed Saber, Control of Chaos in the Burke-Shaw system of fractal-fractional order in the sense of Caputo-Fabrizio, *J. Appl. Math. Comput. Mech. *(2023).

[49] Najat Almutairi & Sayed Saber, Application of a time-fractal fractional derivative with a power-law kernel to the Burke-Shaw system based on Newton's interpolation polynomials. *MethodsX*, 102510 (2023). 10.1016/j.mex.2023.102510 PMC10784698 38223217

[50] Caputo, M. & Fabrizio, M. A new definition of fractional derivative without singular kernel. *Prog. Fract. Differ. Appl.* **1**, 73–85 (2015).

[51] Caputo, M. & Fabrizio, M. On the notion of fractional derivative and applications to the hysteresis phenomena. *Meccanica* **52**(13), 3043–3052. 10.1007/s11012-017-0652-y (2017).

